# 3q27.1 microdeletion causes prenatal and postnatal growth restriction and neurodevelopmental abnormalities

**DOI:** 10.1186/s13039-022-00587-0

**Published:** 2022-03-03

**Authors:** Subit Barua, Elaine M. Pereira, Vaidehi Jobanputra, Kwame Anyane-Yeboa, Brynn Levy, Jun Liao

**Affiliations:** 1 Department of Pathology, Anatomy, and Laboratory Medicine, West Virginia University Health Sciences Center, Morgantown, WV USA; 2 Division of Clinical Genetics, Department of Pediatrics, Columbia University Medical Center, New York, NY USA; 3 Department of Pathology and Cell Biology, Columbia University Medical Center, New York, NY USA

**Keywords:** 3q27.1 microdeletion, *DVL3*, *AP2M1*, *PARL*, CNV interpretation

## Abstract

**Background:**

Overlapping microdeletions of chromosome 3q26-3q28 have been reported in eight individuals. The common phenotype observed in these individuals include intrauterine growth restriction, short stature, microcephaly, feeding difficulties, facial dysmorphisms, limb abnormalities and developmental delay. The most striking clinical features shared among all reported cases is prenatal and postnatal growth restriction and neurodevelopmental abnormalities.

**Case presentation:**

We identified two additional individuals with overlapping deletions and shared clinical features by high-resolution SNP oligonucleotide microarray, and refined the smallest region of overlap (SRO) to a 1.2 Mb genomic location in chromosome 3q27.1 by reviewing and comparing all published cases. We evaluated the SRO using ACMG/ClinGen current recommendations for classifying copy number variants (CNVs), and discussed the contribution of the genes deleted in the SRO to the abnormal phenotype observed in these individuals.

**Conclusions:**

This study provides further evidence supporting the existence of a novel 3q27.1 microdeletion syndrome and suggests that haploinsufficiency of potential candidate genes, *DVL3*, *AP2M1*, and *PARL* in the SRO in 3q27.1 is responsible for the phenotype.

**Supplementary Information:**

The online version contains supplementary material available at 10.1186/s13039-022-00587-0.

## Background

Intrauterine growth restriction (IUGR) is a condition where fetal growth did not achieve the normal growth expected for the gestational age. It is a leading cause of perinatal mortality and morbidity that needs long-term follow up due to an increase risk for future development of chronic diseases. IUGR can be caused by genetic, epigenetic, metabolic, endocrine, or environmental factors [1–4]. Symmetric IUGR, where weight, length, and head circumference are equally affected, can be secondary to infections, chromosomal variants, or the suboptimal nutritional supply that affect the pregnancy at early stages. Asymmetric IUGR, where the weight is most affected and the head circumference is spared, occurs more commonly at later stages of pregnancy and is commonly from lack of nutrition. When IUGR is identified, the expectant mother is worked up for infectious through TORCH titers and are normally offered invasive testing with a microarray and chromosome analysis to rule out common causes of IUGR. Specifically, there is a strong association of IUGR with chromosomal aberrations. In some micro-duplication/deletion syndromes, IUGR is a major and only manifestation [5, 6].

One of the chromosome regions associated with IUGR is located in chromosome 3q26-3q28. Patients with microdeletions in this region are rare and not well described. To date eight cases have been reported in the literature with deletions varying in size from ~ 2.0 to 8.4 Mb [7–12]. Although their breakpoints are not recurrent, these patients share an apparently distinct phenotype including IUGR, microcephaly, short stature, facial abnormities, and feeding difficulties. However, the clinical significance and genetic mechanism of the 3q26q28 microdeletion are not fully established. Here we report two unrelated individuals harboring overlapped microdeletions in this region and sharing clinical features with those reported in the literature. Using high-resolution single nucleotide polymorphism (SNP) microarrays, we narrowed the Smallest Region of Overlap (SRO) to a size of 1.2 Mb at chromosomal band 3q27.1. This SRO region contains 46 genes, 24 of which are OMIM-annotated and eight of which are associated with disease (*KLHL24*, *EIF2B5*, *DVL3*, *AP2M1*, *ALG3*, *EIF4G1*, *CLCN2*, and *THPO*).

Genome-wide assessment of copy-number variants (CNVs) is widely applied to assess the clinical significance of pre- and post-natal congenital abnormalities. However, in-between clinical laboratories, the assessment of CNV classification remains inconsistent due to lack of uniform scoring metrics. To assist clinical laboratories in the accurate and consistent classification of reporting CNVs, ACMG and ClinGen recently published technical standards for CNV interpretation [13]. We applied their recommended quantitative and evidence-based scoring framework to evaluate the deduced 1.2 Mb SRO at chromosomal band 3q27.1 and classified it as a pathogenic deletion.

## Case presentation

### Proband-1

A G1P0 woman with naturally conceived male fetus was referred for prenatal diagnosis due to second trimester abnormal ultrasound findings (Table 1): < 5% abdominal circumference, absent nasal bone, placentomegaly and severe oligohydramnios. Fetal measurement revealed a fetal weight at less than the tenth centile, which was consistent with a diagnosis of IUGR. Fetal echocardiogram was concerning for cardiomegaly and hypoplastic aortic arch. The proband was born at 37 weeks and 3 days of gestation via normal spontaneous vaginal delivery with a birth weight of 1785 g. Postnatal echocardiogram ruled out any cardiac anomaly and newborn screening was normal. By 3 months old, the proband continued to have growth parameters below the 5th centile; weight:3.7 kg (< 3rd centile); length: 54.5 cm (< 3rd centile); occipitofrontal circumference (OFC) 38.5 cm (5th centile). He was dysmorphic with microcephaly, mild frontal bossing, bilateral epicanthal folds, hypotelorism, posteriorly rotated ears, flat nasal bridge, micrognathia, high arched palate, left palmer simian crease, and increased muscle tone. By 18 months old he was receiving speech and feeding therapies. Gross motor skills were delayed as he was only beginning to cruise. By 30 months old, he started to walk, and by 3 years old be had 20–30 words. By 3 years of age, the proband started to walk. A neuropsychological evaluation at 4yo demonstrated the proband was too cognitively limited to complete the exam. The proband’s family was not interested in a gastrostomy tube, and he continued to grow at < 3rd percentile though he was not a picky eater. By 6 years old, the proband has not been potty trained. At the last evaluation at 8 years old, he continues to be in special education class with services and continues to have trouble with comprehension. His physical exam still demonstrated < 2nd percentile for height, weight, and head circumference. Additional findings of dolichocephaly and arachnodactyly were also noted.

**Table 1:** Summary of clinical and genetic features of ten previously reported and present cases with 3q26-3q28 microdeletions

	Mandrile et al. [7]			Zarate et al. [8]	Dasouki et al. [9]	Sahin et al. [10]
	Patient 1	Patient 2	Patient 3			
Chromosomal regions (hg19)	3q26.33-3q27.2 (chr3:181,648,378–185,786,898)	3q26.33-3q27.2 (chr3:181,692,255–185,969,168)	3q27.1-3q27.2 (chr3:183,047,473–185,140,522)	3q26.33-3q27.5 (chr3:182,189,525–187,212,935)	3q26.33-3q27.1 (chr3:182,470,516–184,469,308)	3q26.33-3q27.3 (chr3:182,507,317–186,845,923)
Size of deletion	4.14 Mb	4.28 Mb	2.09 Mb	5 Mb	2 Mb	4.3 Mb
Inheritance	De novo	De novo	Unknown (not maternal)	Unknown	De novo	Unknown
Sex	Male	Male	Female	Female	Male (47,XXY)	Female
Age at last examination	6 years	18 years	12 years	16 years	9.5 years	7 years
Oligohydramnios	No	No	No	No	No	Yes
Intrauterine growth restriction	Yes	Yes	Yes	Yes	Yes	Yes
Short stature	Yes	Yes	Yes	Yes	Yes	Yes
Feeding problems	Yes	Yes	Yes	Yes	No	No
Microcephaly	Yes	Yes	No	Yes	Yes	Yes
Cognitive abnormalities	Developmental delay, severe intellectual disability	Developmental delay, severe intellectual disability	Developmental delay, learning disability, borderline IQ	Developmental delay, intellectual disability	Developmental delay	Developmental delay, intellectual disability
Behavioral abnormalities	No	Hyperactivity	ADHD, an extremely friendly personality	No	Asperger syndrome	No
Seizure	No	Tonic seizure at birth	No	Tonic–clonic and myoclonic photo-convulsive seizures	No	No
Hypotonia	Yes	Yes	Yes	Yes	Yes	No
Facial dysmorphisms	Yes	Yes	Yes	Yes	Yes	Yes
Hands abnormalities	Clinodactyly (4th finger)	No	Mildly tapered fingers with flattening of the ulnar borde	Clinodactyly (5th finger)	No	No
Feet abnormalities	Pes planus, third toes overlap with fourth toes	Pes planus, abnormal foot position	Mild pes planus	Pes planus, overlapping toes	No	No
Dental abnormalities	Yes	Yes	Yes	Yes	Yes	No
Heart defects	Patent ductus arteriosus	No	No	No	No	Patent foramen ovale, mild pulmonary hypertension
Other findings	Recurrent upper airway infections, inguinal hernia, mild kyphosis and pectus carenatum, joint laxity	Recurrent otitis media, conductive hearing loss, delayed puberty	Recurrent otitis media, astroesophageal reflux, multiple freckles of the left forearm and café au lait spot of left lower flank, pre-diabetes	Sensorineural hearing loss, severe gastroesophageal reflux, mild generalized hypertonia at 16	Thrombocytopenia, recurrent infections, easy bruising, tremors	Diaphragm evantration, irregular respiration and tachypne, bilateral segmental perfusion defects

	Bouman et al. [11]	Ounap et al. [12]	This study		Consensus
			Proband-1	Proband-2	
Chromosomal regions (hg19)	3q26.33-3q27.3 (chr3:183,220,510–189,409,266)	3q26.33-3q28: (chr3:182,674,821–191,025,402)	3q27.1-3q28 (chr3:183,011,106–187,947,036)	3q27.1-3q27.2 (chr3:182,950,371–185,324,970)	Smallest region of overlap: 3q27.1 (chr3:183,220,510–184,469,308)
Size of deletion	6.18 Mb	8.35 Mb	4.93 Mb	2.37 Mb	1.2 Mb
Inheritance	De novo	Unknown	De novo	Unknown (not maternal)	5 De novo, 5 unknown
Sex	Female	Female	Male	Female	4 Male and 6 Female
Age at last examination	Fetus of 22 + 1 weeks	16 years	8 years	3 years	Age range: Fetus of 22 + 1 weeks-18 years
Oligohydramnios	No	No	Yes	Yes	3/10
Intrauterine growth restriction	Yes	Yes	Yes	Yes	10/10
Short stature	N/A	Yes	Yes	Yes	9/9
Feeding problems	N/A	Yes	Yes	Yes	7/9
Microcephaly	Yes	Yes	Yes	Yes	8/10
Cognitive abnormalities	N/A	Developmental delay, mild Intellectual disability	Developmental delay, intellectual disability	Developmental delay	9/9
Behavioral abnormalities	N/A	Tics and nail biting	No	No	4/9
Seizure	N/A	No	No	No	2/9
Hypotonia	N/A	Yes	No	No	6/9
Facial dysmorphisms	Yes	Yes	Yes	Yes	10/10
Hands abnormalities	Bilateral clinodactyly (5th finger)	Clinodactyly (5th finger)	Arachnodactyly	No	6/10
Feet abnormalities	Bilateral club feet	Mild left Club foot	No	No	6/10
Dental abnormalities	N/A	Yes	Yes	N/A	7/8
Heart defects	Atrial septal defect, coarctation	Supravalvular aortic and pulmonary stenosis	No	No	4/10
Other findings	Clitoromegaly	Brain atrophy in frontal lobe, sensorineural hearing loss, hypertonia and increased deep tendon reflexes at 16	Hypertonia since 3 months, dolichocephaly	Hyperbilirubinemia	Various

### Proband-2

The proband is a female child of non-consanguineous Dominican desecnt. Prenatal ultrasounds at 24 weeks gestational age was concerning for symmetric IUGR where the head circumference was 3 standard deviations below the mean. An infectious and cardiac workup were normal. At 37 weeks gestational age oligohydramnios was noted. The proband was born full term as small for gestational age with weight, length and head circumference all < 2nd percentile (weight 1.725 kg, length 44 cm, head circumference 30 cm). A post-natal TORCH panel was sent along with CMV urine analysis which were normal. She passed her newborn screen. Though the proband was discharged within 48 h, she was readmitted for hyperbilirubinemia that required phototherapy. At 6 months of age, her growth parameters were only at the 1st percentile. She continued to have failure to gain weight, prompting an admission for further workup when she was 11 months old. A nasogastric tube was placed to help with her feedings. During the admission she had a genetics evaluation. Her exam was notable for microcephaly, epicanthal folds, hypertelorism, an arched palate, and retrognathia. After her hospital discharge, the proband’s family had difficulty with follow up involving therapies and sub-specialty providers. She qualified for speech therapy but was not able to attend many of the sessions. Although she qualified for physical therapy, the family declined services once she began to walk at 20 months of age. At 3 years old she has many words but is working on sentences. She remains consistently at less than the 3rd percentile for height, weight, and head circumference. At this time, the patient is constantly encouraged to follow up with services through the school system, and to schedule appointments with appropriate subspecialty providers to help with feeding issues.

## Results

### Proband-1

SNP Oligonucleotide Microarray Analysis (SOMA) using Affymetrix GeneChip Human Mapping 6.0 SNP array and Affymetrix Chromosome Analysis Suite 3.3 (Affymetrix, Santa Clara, CA) revealed that Proband-1 harbors a 4.93 Mb deletion in genomic coordinates 183,011,106–187,947,036 (hg19) corresponding to chromosomal bands 3q27.1q28 (arr[hg19] 3q27.1q28(183,011,106_187,947,036)x1; Additional file 1: Fig. S1a). This deleted region contains 122 genes, 53 of which are OMIM-annotated and 20 of which are associated with disease. Maternal SOMA showed that the mother does not have the same deletion in the long arm of chromosome 3 (Additional file 1: Fig. S1b). FISH using a BAC probe RP11-919L13 further confirmed that this deletion is present in the proband but not in the father (Additional file 1: Fig. S1c and d) and is therefore de novo in origin. No other CNVs except for those commonly seen in normal populations were detected in this individual.

### Proband-2

A karyotype analysis of Proband-2 with a resolution level of 525 bands revealed a normal female chromosome complement (46,XX). SOMA in Proband-2 identified a 2.37 Mb deletion (Additional file 1: Fig. S2a) in the chromosomal region 3q27.1q27.2, corresponding to genomic coordinates 182,950,371–185,324,970 (arr[hg19] 3q27.1q27.2(182,950,371_185,324,970)x1). This deleted region contains 66 genes, 30 of which are OMIM-annotated and 10 of which are associated with disease. FISH using a BAC probe RP11-919L13 confirmed the presence of this deletion in Proband-2 and excluded its maternal inheritance (Additional file 1: Fig. S2b and c). Father is unavailable for testing. No other CNVs except for those commonly seen in normal populations were detected in this individual.

## Discussion and conclusions

In the literature, there are eight previously reported cases carrying 3q26-3q28 microdeletions with sizes of 2–8.4 Mb that overlapped with the deleted chromosomal regions in two patients from this study [7–12]. The clinical phenotype of individuals with 3q26-3q28 microdeletions is heterogeneous: IUGR, postnatal growth impairment, feeding problems, short stature, dysmorphic facial features, microcephaly, seizure, dental and limb abnormalities, developmental delay, intellectual disability, hypotonia, and thrombocytopenia. Additionally, in the Decipher database, several individuals harboring the deletions (range of 1.8–2.7 Mb) in this region has been reported to exhibit variety of phenotypes including intrauterine growth retardation, short stature, microcephaly, facial dysmorphisms, hypotonia, developmental delay, and cardiac defects (DECIPHER ID: 317983, 276986, and 323724). While there is some degree of phenotypic variability that primarily relates to the size of the deletion, the most striking clinical features shared among all reported cases are prenatal and postnatal growth restriction, as well as neurodevelopmental abnormalities. The clinical presentation of two patients described in this study supports the clinical profile described for other individuals in the literature (Table 1). The genotype–phenotype correlations for loss of the 3q26q28 region are, however, restricted by the fact that these individuals do not share common break points, like those generated in recurring pathogenic CNVs flanked by segmental duplications. Nonetheless, comparison of the clinical and molecular findings in Proband-1 and Proband-2 with the previous reported individuals suggests that this is a microdeletion syndrome with shared clinical features. Though the precise size and position of these deletions are uncertain, it has been proposed that haploinsufficiency of dosage sensitive genes leads to defined clinical sequelae [7].

**Fig. 1:**
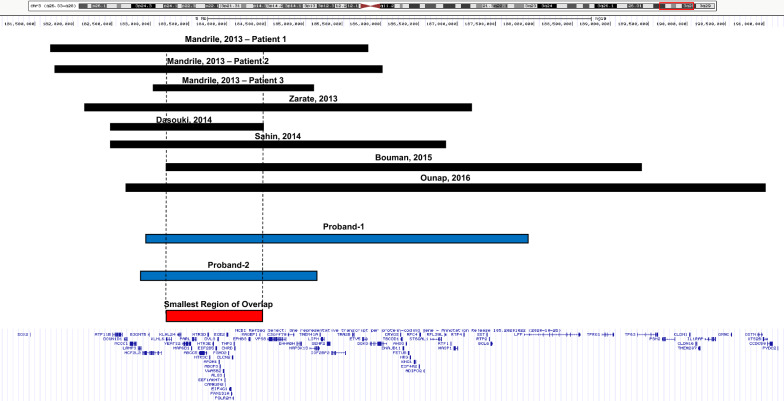
Schematic representation of chromosome 3q26-3q28 region showing previously reported deletions (black), deletions described in this study (blue), and smallest region of overlap (red)

By comparing microarray findings of these ten cases, we mapped the SRO to a size of 1.2 Mb, corresponding to genomic coordinates 183,220,510–184,469,308 (hg19) at chromosomal band 3q27.1 (Fig. 1). Deletions overlapping with this region are absent from population databases including gnomAD SVs v2.1 (controls) and DGV Gold Standard [14, 15]. This SRO region contains 46 genes, 24 of which are OMIM-annotated and eight of which are associated with disease (*KLHL24*, *EIF2B5*, *DVL3*, *AP2M1*, *ALG3*, *EIF4G1*, *CLCN2*, and *THPO*) (Additional file 2 Table S1). Among these genes, *DVL3* is the most interesting one. Heterozygous pathogenic variants in the *DVL3* gene have been associated with autosomal dominant type III Robinow syndrome (MIM#: 616894), which shares many clinical features with the 3q26q28 microdeletion syndrome: short stature (9/9), facial dysmorphic features (10/10), teeth abnormalities (7/8), and hand abnormalities (6/10). Emerging data suggest *DVL3* is a core component in the routing and transmission of canonical and non-canonical Wnt signalasome [16]. In murine, *DVL3* has been detected to express ubiquitously at E7.5, but shortly after it showed elevated expression in heart, CNS, notochord, dorsal root ganglia, branchial arches, limb buds, and somitic mesoderm [17, 18]. These findings further strengthen the role of *DVL3* in diseases by modulating Wnt signaling that is involve in cell migration and tissue morphogenesis in vertebrate development. Indeed, *Dvl3* knock out mice demonstrated partial lethality, conotruncal defects and neural tube defects, including abnormalities in cochlear cells [19]. However, all current known pathogenic variants of *DVL3* are frameshift small insertions/deletions or splice variants in the last two exons; and larger intergenic deletions of *DVL3* have not been described previously [20–22]. Furthermore, it was demonstrated by expression studies that truncating *DVL3* variants escape nonsense-mediated decay (NMD), suggesting a dominant-negative or gain-of-function disease mechanism [21–24]. Therefore, the exact contribution from loss of *DVL3* to phenotype caused by 3q26-3q28 microdeletions is still uncertain at this time.

Beside *DVL3*, the role of *AP2M1* (MIM#: 601024) in developmental delay (9/9), hypotonia (6/9), and seizures (2/9), as well as the role of *PARL* (MIM#: 607858) in growth restriction (10/10) are of great interest. *AP2M1* has recently been associated with impaired intellectual development, poor speech, and delayed walking [25]. Though a recurrent missense variant in *AP2M1* has been reported, *AP2M1* is highly intolerant to loss-of-function variant in general population with a probability of intolerance to loss of function (pLI) of 1.0 and the Haploinsufficiency Score of 8.13. Previous studies with Parl knock out mouse model have shown that *Parl* plays an essential physiological role in the neurological homeostasis [26], and *Parl* deficiency results in growth retardation, cachexia, and severe atrophy of skeletal muscle, thymus, and spleen [27]. However, we think the growth phenotype caused by this SRO is predominantly overlap with *DVL3* related Robinow syndrome and further study is warranted to associate the role of *PARL* in this phenotype. The remaining OMIM genes in the SRO (*ALG3*, *CLCN2*, *EIF2B5*, *EIF4G1*, *KLHL24*, and *THPO*) are associated with autosomal recessive conditions and therefore are less likely to have major contributions to these patients’ phenotype.

In order to evaluate the clinical significance of the SRO, we further assessed the deduced SRO corresponding to genomic coordinates, chr3:183,220,510–184,469,308 (hg19) using ACMG/ ClinGen current recommendations for classifying copy number variants (CNVs) [13]. This SRO harbors 26 protein coding RefSeq genes (Criteria 3B, points given: 0.45). Three of them, *PSMD2*, *AP2M1*, and *EIF4G1* are predicted to be haploinsufficiency genes by the gnomAD pLI score [28], the gomAD LOEUF score [29], and the DECIPHER HI index [30] (Additional file 2: Table S1; Criteria 2H, points given: 0.15).. To the best of our knowledge, in the literature the SRO overlap with four previously assumed (due lack of molecular confirmation for paternity and maternity) de novo cases (individuals 1, 2, 5, and 7 in Table 1) with phenotype that is consistent with the gene/genomic region, but not highly specific and/or with high genetic heterogeneity (Criteria 4C, points given: 0.40). Moreover, observed copy number loss is assumed de novo (due lack of molecular confirmation for paternity and maternity) for Proband-1 in this study (Criteria 5A, points given: 0.1). Using these recommendations as a framework, we classified the SRO as pathogenic (Total score: 1.1).

In conclusion, in this study we present two additional individuals with phenotype similar to previously reported cases with overlapped deletions in chromosome 3q26q28 region. It provides further evidence supporting the existence of this novel microdeletion syndrome. Additionally, our molecular cytogenetic and clinical findings defined the 1.2 Mb SRO at chromosomal band 3q27.1 as the critical region for this microdeletion syndrome. The refinement of this critical region suggests that deletion of at least three genes (*DVL3*, *PARL* and *AP2M1*) may contribute to anomalies observed in these individuals. At last, we utilized the new ACMG/Clingen standards for CNV interpretation with refined molecular mapping that improved our ability for clinical diagnosis and genetic counselling of individuals harboring similar imbalance.

## Supplementary Information

**Additional file 1: Figure S1.** Test results for Proband-1 and his parents. **a** SOMA result showing the 3q27.1q28 deletion in the Proband-1. **b** Normal SOMA result from the mother. **c** FISH result using the BAC probe RP11-919L13 confirmed the presence of the deletion in Proband-1. **d** Normal FISH result from the father. **Figure S2.** Test results for Proband-2 and her mother. **a** SOMA result showing the 3q27.1q27.2 deletion in Proband-2. **b** FISH result using the BAC probe RP11-919L13 confirmed the presence of the deletion in Proband-2. **c** Normal FISH result from the mother (PPTX 714 KB)

**Additional file 2: Table S1.** Summary of protein coding genes within the 1.2 Mb in chromosome 3q27.1 (XLSX 13 KB)

## Data Availability

The datasets used and/or analysed during the current study are available from the corresponding author on reasonable request.

## Abbreviations

CNV: Copy number variants
IUGR: Intrauterine growth restriction
NMD: Nonsense-mediated decay
OFC: Occipitofrontal circumference
SNP: Single nucleotide polymorphism
SOMA: Single nucleotide polymorphism oligonucleotide microarray analysis
SRO: Smallest region of overlap
