# Supplementary material for: 3q27.1 microdeletion causes prenatal and postnatal growth restriction and neurodevelopmental abnormalities
Source: Mol Cytogenet. 2022 Mar 3;15:7. doi: 10.1186/s13039-022-00587-0 (PMC8895857; doi:10.1186/s13039-022-00587-0)
Supplement: Supplementary file 1 — Additional file 1: Figure S1. Test results for Proband-1 and his parents. a SOMA result showing the 3q27.1q28 deletion in the Proband-1. b Normal SOMA result from the mother. c FISH result using the BAC probe RP11-919L13 confirmed the presence of the deletion in Proband-1. d Normal FISH result from the father. Figure S2. Test results for Proband-2 and her mother. a SOMA result showing the 3q27.1q27.2 deletion in Proband-2. b FISH result using the BAC probe RP11-919L13 confirmed the presence of the deletion in Proband-2. c Normal FISH result from the mother (PPTX 714 KB) [file 13039_2022_587_MOESM1_ESM.pptx]

## Slide 1
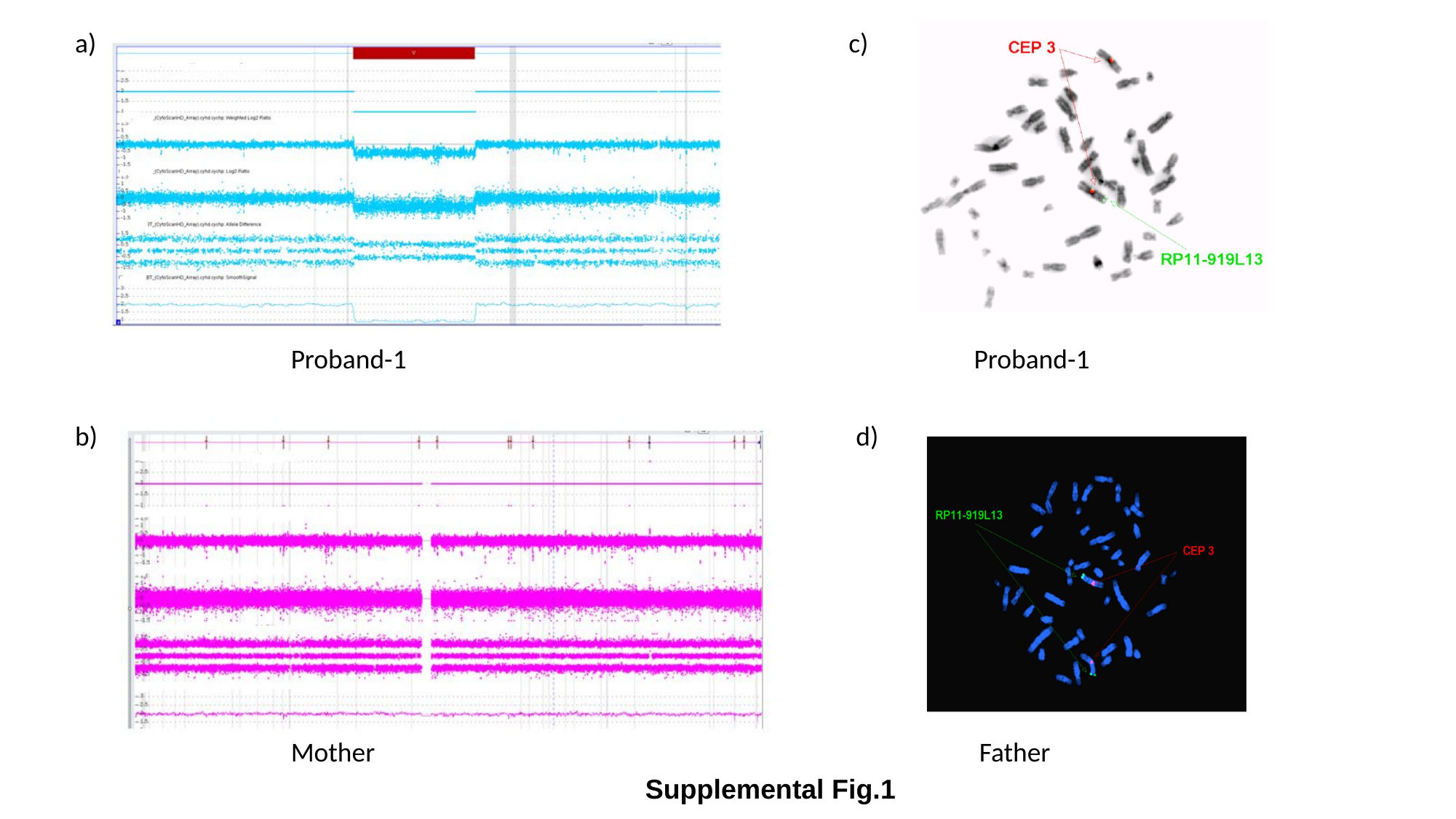

a) c)
b) d)
Proband-1 Proband-1
Mother Father
Supplemental Fig.1

## Slide 2
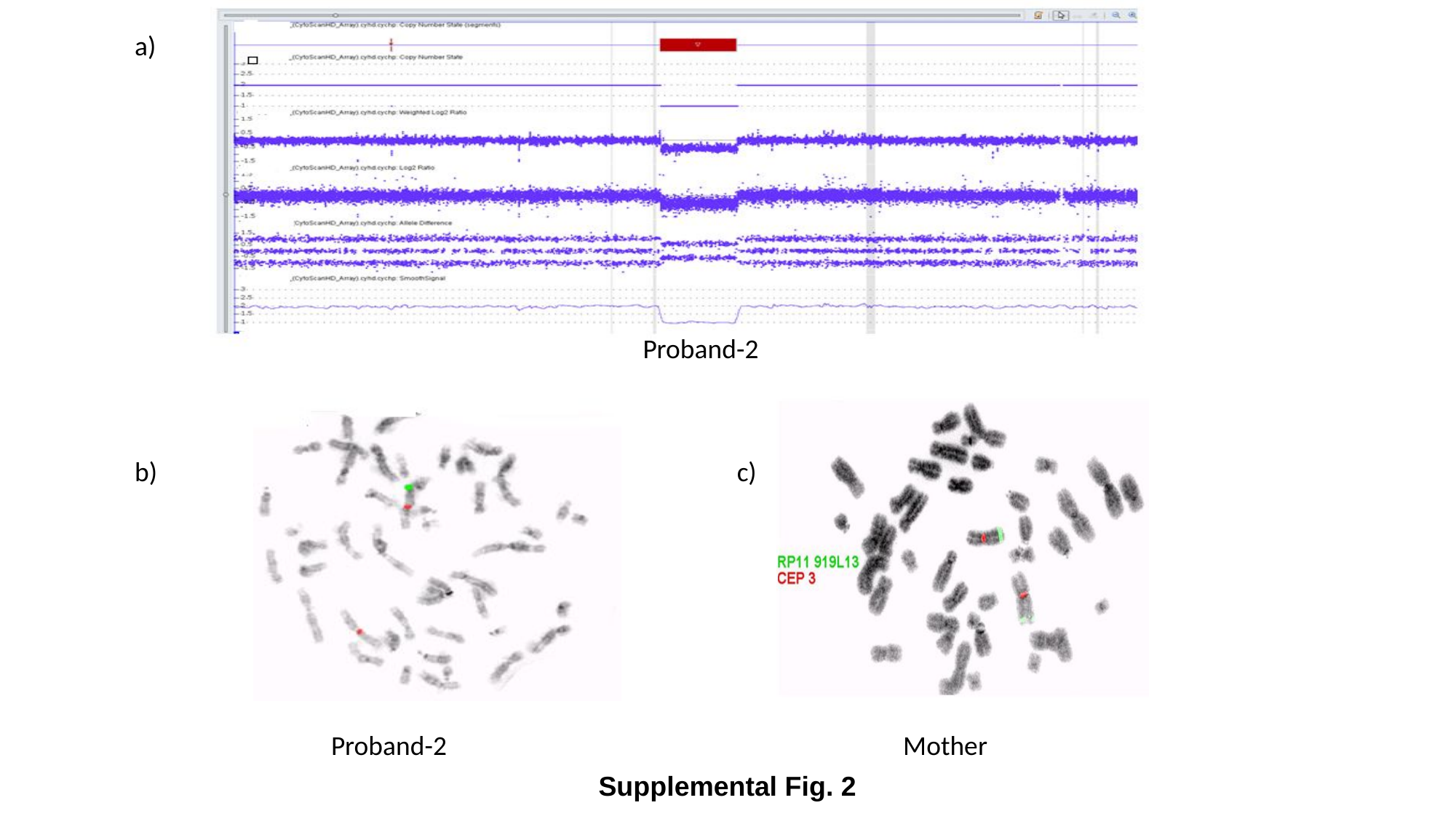

a)
b) c)
Proband-2
Proband-2 Mother
Supplemental Fig. 2
